# Suture-Mediated Intermediate and Terminal Closure When Using the Aortix Percutaneous Mechanical Circulatory Support Device

**DOI:** 10.1016/j.jscai.2023.101038

**Published:** 2023-05-19

**Authors:** Matheen A. Khuddus, Mir B. Basir, Aaron Palmer, Av Edidin

**Affiliations:** a The Cardiac and Vascular Institute, Gainesville, Florida; b Division of Cardiology, Henry Ford Hospital, Detroit, Michigan; c Product Development, Procyrion, Inc, Houston, Texas

**Keywords:** hemostasis, large-bore closure, percutaneous, suture-mediated

## Abstract

**Background:**

The Aortix percutaneous mechanical circulatory support (pMCS) 18F micromechanical axial impeller–driven pump is percutaneously placed and retrieved from the descending aorta above the renal arteries. Pump deployment and retrieval use defined steps to allow suture-mediated closure around the exiting power lead and for terminal hemostasis after device retrieval after therapy. The overall procedure was validated preclinically in ovine and porcine models and has since proven reliable in a recently completed IDE feasibility study (NCT04145635). This study describes the steps of a novel technique for reliable suture-mediated vessel closure when using the Aortix pMCS device.

**Methods:**

The principal steps associated with the procedure comprised ultrasound-guided access of the femoral artery, preclose placement of 4 Perclose (Abbott) sutures, achievement of intermediate hemostasis by locking 3 of the 4 sutures around the 6F exiting power lead, breaking the 3 locked sutures to permit vessel reentry for device removal, and finally, vessel closure using the fourth preclose suture along with 1 or more postclose suture(s) to close the femoral artery using a hybrid dual-sheath technique.

**Results:**

The standardized steps described have been developed and used over the course of 21 clinical cases by 12 operators who were initially procedurally naïve but experienced in large-bore access.

**Conclusions:**

Preclose and postclose suture-mediated closure is a reliable means of managing the exiting power lead of the Aortix pMCS and closing the large-bore arteriotomy after pump retrieval. The steps outlined in this study may have applicability to other procedures requiring large-bore access or using suture-mediated closure.

## Highlights

• Tautening and traveling of Perclose sutures are critical and separate steps when using multiple suture-based vascular access closure devices
• Releasing pressure on the artery is critical to retraveling a suture down to the arteriotomy after dilation or large-bore sheath removal
• The Snared Knot Pusher should be reserved for final tightening and locking steps only; blue rail tension is sufficient to travel the knot down to the artery
• Perclose sutures can be reliably tightened and locked but subsequently broken for removal or replacement

## Central Illustration

**Figure:**
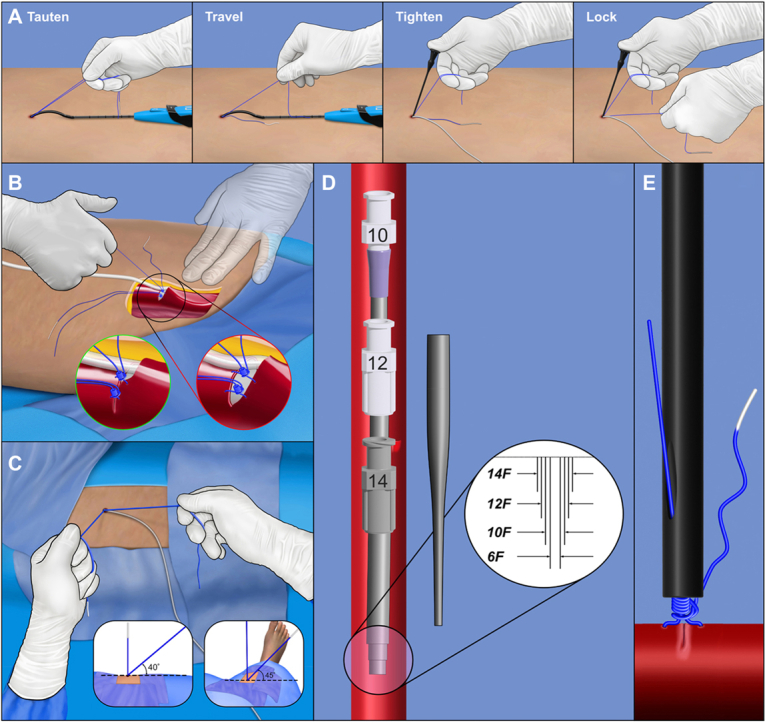

## Introduction

Large-bore (≥16F) femoral artery access is necessary for a number of interventional procedures. Although a few dedicated arterial closure devices exist, preclose placement using at least 2 Perclose devices (Abbott) is an established suture-mediated technique for closure of a large-bore arterial access site.^1^ Alternatively, when a preclose strategy is not used or fails, postclose suture-mediated closure techniques, such as a dualsheath or wire technique, can be used.^2^, ^3^, ^4^, ^5^, ^6^

The Aortix System (Procyrion) is a percutaneous mechanical circulatory support device (pMCS) that is positioned in the descending aorta and designed to treat patients with cardiac and renal impairment. It is an axial impeller–driven pump that is percutaneously delivered and retrieved through the common femoral artery (CFA) using a standard 18F inner diameter/21F outer diameter large-bore sheath. The pump entrains and accelerates blood flow superior to the renal arteries, reducing cardiac workload and increasing renal perfusion. The therapy provided by Aortix, currently under investigation in patients with heart failure with worsening renal function or cardiorenal syndrome in a pilot study (NCT04145635), is indicated for use for ≤7 days.

To deliver and later retrieve the Aortix device, the femoral arteriotomy is percutaneously accessed and closed twice during separate procedures. First, after pump deployment and removal of the introducer sheath, hemostasis is obtained and maintained around the exiting 6F power lead supplying electrical energy to the implanted pump. Second, after ≤7 days of therapy, the vessel is reaccessed for pump removal, and closure is performed again postremoval at the arterial access site. We describe a series of steps using a well-established combination of preclose and postclose suture-mediated techniques to enable the procedure.

To date, the techniques described have been developed and used over the course of 21 clinical cases by 12 operators who were initially procedurally naïve but experienced with large-bore suture-mediated access and closure and/or pMCS use.

## Methods

The Aortix pMCS is an 18F device with a 6F power lead, which directly exits the femoral artery of the patient and is connected to a battery-operated controller to supply power to the device during therapy. The pump is dynamically stabilized in the aorta using atraumatic struts and mechanically tethered at the arteriotomy access site by the suture-mediated closure.

The steps essential to the pump’s deployment include: (1) vessel access with ultrasound guidance; (2) preclosure of the arterial access site; and (3) achieving suture-mediated intermediate hemostasis around the exiting power lead. After ≤7 days of pump therapy the additional steps include: (4) breaking the sutures to permit vessel reentry for device removal and (5) vessel closure.

### Detailed procedure

#### Vessel access

Using ultrasound guidance, the CFA is entered with a micropuncture needle proximal to its bifurcation at the level of the projected center of the femoral head. Contrast or CO_2_ angiography,^7^ using a minimum amount of diluted contrast, can be performed then at the discretion of the operator. After advancing an 80- to 150-cm J-tip access wire through the micropuncture sheath into the vasculature, a generous tissue tract is created to allow space for 4 sutures and a 6F power lead by making a 1 cm skin incision and using a curved Kelly to bluntly dissect the tract. Then, a 6F sheath is advanced over the wire into the CFA as per standard practice.

#### Preclosure of arterial access site

Four Perclose sutures are delivered down to the artery in a preclose fashion—3 are used to obtain intermediate hemostasis over the power lead, whereas the fourth is used to begin arterial wall approximation and terminal closure after pump removal. In addition, the fourth suture can be engaged to assist in power lead hemostasis should one of the other 3 prove ineffective.

**Figure 1:**
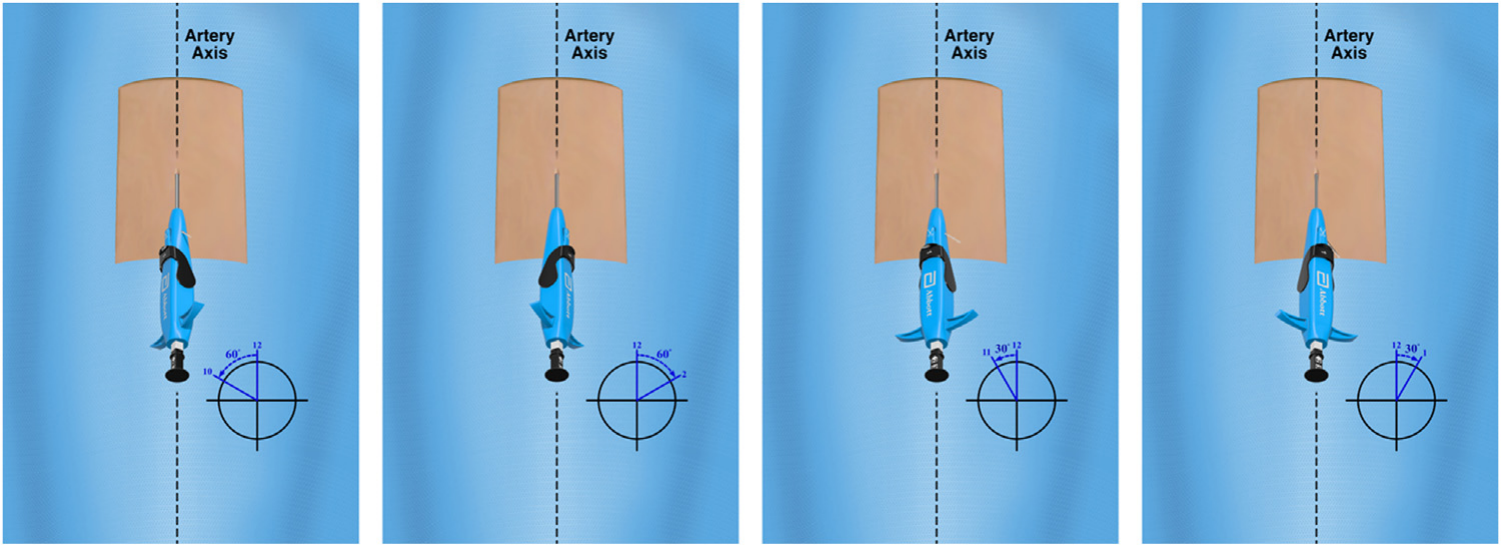
**Instrument positioning and clocking of the 4 preclose Perclose sutures**.

**Figure 2:**
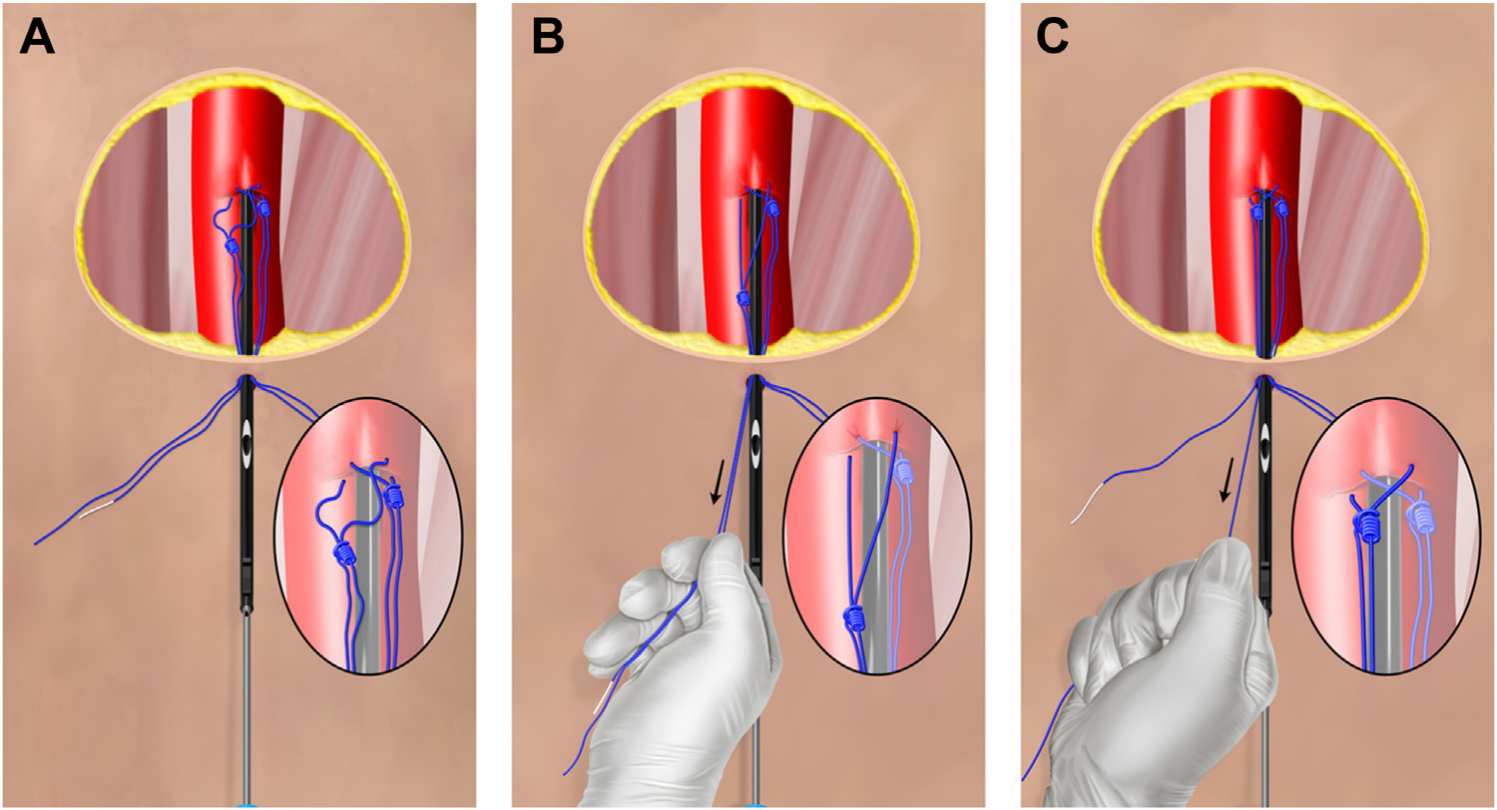
**Visualizing tautening and traveling.** (**A**) The 10-o’clock suture has been deployed, and the 2-o’clock suture has just been placed. (**B**) The operator tautens and centralizes the suture by pulling on both limbs with 1 hand. (**C**) Finally, the newly placed knot is traveled down to the artery by applying tension only to the blue (rail) limb.

The 4 Perclose sutures are clocked at the 10-, 2-, 11-, and 1-o’clock positions relative to the vessel axis, which is the 12-o’clock reference position (Figure 1). Adherence to the standard Perclose suture deployment steps is recommended—in particular, following suture release from the delivery instrument, each suture should be tautened by simultaneously pulling on both limbs to centralize the suture and traveled by pulling only the blue (rail) limb (Figure 2). Once each suture is traveled to the artery by blue limb railing, the limbs are collated using atraumatic clips—the SuperClip (Advanced Vascular Dynamics) has been our method of choice. Hemostats are not used to gather or collate sutures because they can cause stress risers in the filament, which can impede subsequent suture breakage at the time of pump retrieval.

**Figure 3:**
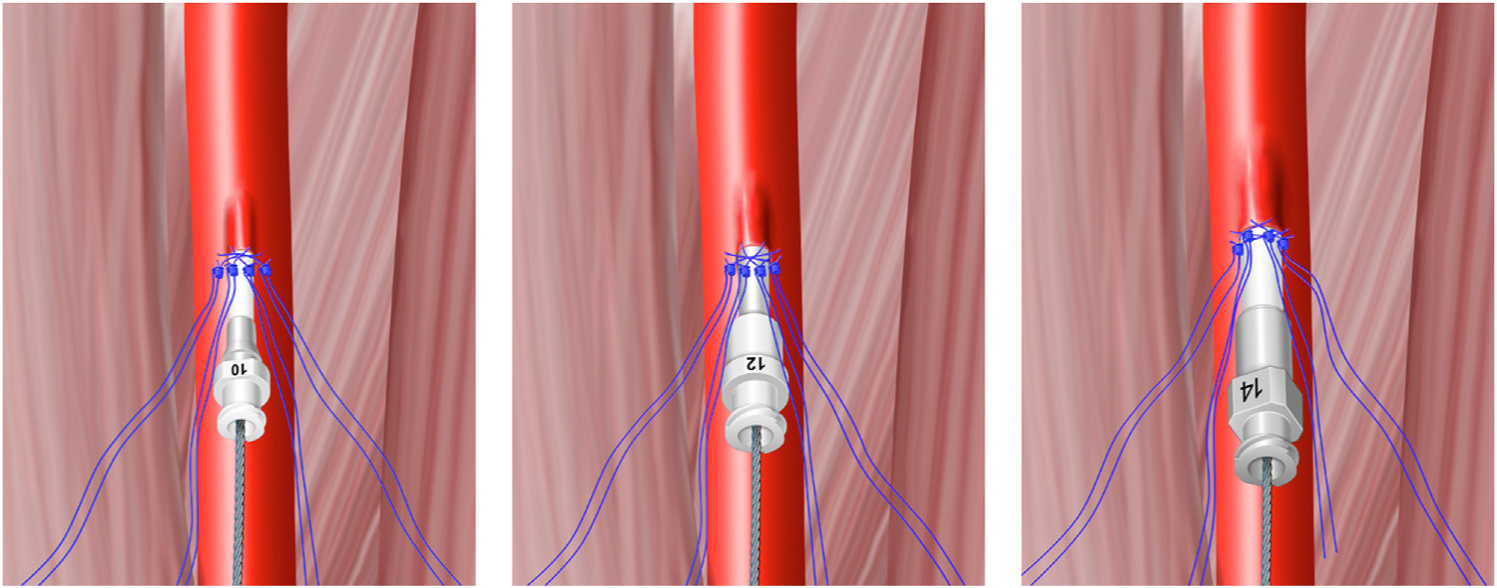
**Serial dilation to reopen the previously traveled sutures**.

After the 4 sutures have been preclosed and traveled down to the artery, the artery is serially dilated using 10F, 12F, and 14F dilators (Coons Taper has been our preferred dilator) whose geometry facilitates gradual reopening of each suture by virtue of the dilators’ long taper (Figure 3). Because each suture is a cinch knot, it can be repeatedly traveled to the artery and redilated as the procedure requires. After sequential dilation, the delivery sheath with its tapered dilator may be easily passed into the vasculature.

#### Intermediate hemostasis

Once the Aortix pump is deployed in the descending aorta the large-bore sheath is removed. Manual pressure is used to control the artery with the 6F power lead exiting the arteriotomy. To achieve intermediate hemostasis, each suture is retraveled back to the artery using blue limb tension (railing)—the knot pusher is not used in this step. Pressure on the artery should be momentarily relaxed in tandem with advancement of the knot back to the artery.

**Figure 4:**
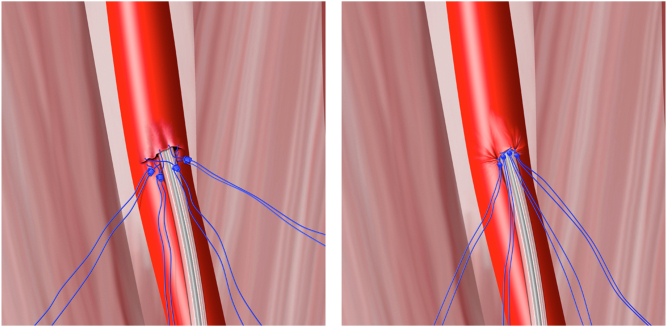
**Mechanical intermediate hemostasis over the exiting power lead can be rapidly achieved by retraveling each suture down to the artery after large-bore sheath removal.** Appearance after sheath removal (left) and the following retravel (right).

The sutures are retraveled in the order placed; the degree of hemostasis is assessed while maintaining blue limb tension for each suture in turn. All 4 sutures are typically retraveled in sequence twice. Each suture’s limbs are recollated immediately after travel to ensure that no confusion arises as to which limbs belong to which clocked suture (Figure 4).

**Figure 5:**
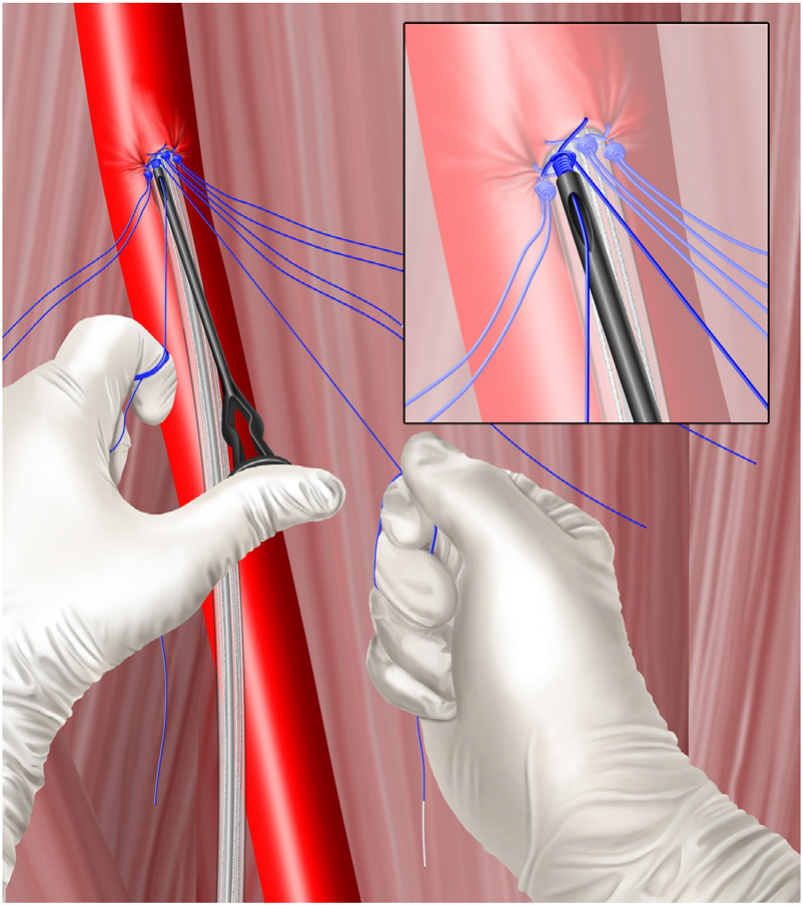
**Technique for tightening and locking knot over exiting power lead**: compression on knot pusher and tension on blue limb with one hand; then, suture is locked with pull on white limb.

Finally, once intermediate hemostasis is clearly apparent during suture travel and blue limb tension, the 10-, 2-, and 11-o’clock sutures are tightened and locked using the polymeric knot pusher (Snared Knot Pusher) because we found that the proprioception afforded by this tool superior to that of the metal shaft combination pusher/cutter, which we tend to discard in any case at the outset to avoid the possibility of inadvertently cutting the suture limbs as would typically be performed. Knot tightening is performed with the thumb pressing downward on the knot pusher while the blue limb is maintained under tension using the remaining fingers of the same hand, allowing knot cinching and crushing simultaneously. Then, a gentle pull on the white (nonrail) limb will lock the suture (Figure 5).

After locking the 3 sutures providing intermediate hemostasis, the respective suture limb pairs are threaded into individual 1.0- to 3.0-mL syringes and the plunger inserted to keep the assembly intact.

#### Suture breaking and vessel reentry at the time of pump removal

Before vessel reentry, the retrieval sheath/dilator combination is advanced right up to the arteriotomy site. Then, the 3 locked sutures are broken in the reverse order of placement—11-, 2-, and, finally, 10-o’clock sutures are broken.

**Figure 6:**
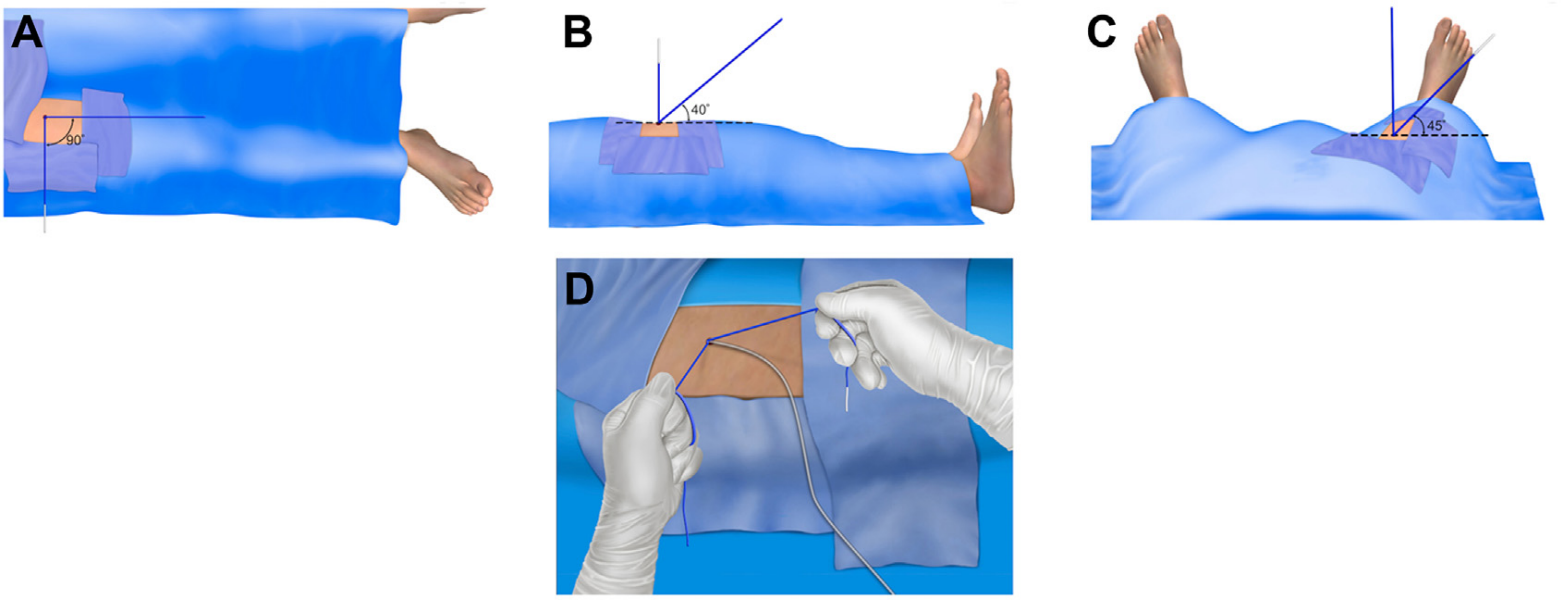
**Hand positioning and overall view of suture breaking.** (**A**) Limbs are first oriented at a right angle; (**B**) the blue rail limb is elevated to approximately the same angle as the initial access into the vessel; (**C**) the white nonrail limb is elevated to 45°; and (**D**) the overall appearance of the hands just before snapping off the white limb.

A suture may be readily broken by following the alignment steps and hand positions shown in Figure 6. Basically, tension is placed on the blue limb when it is placed at the same angle relative to the skin as was the original Perclose instrument (axis of access), and the white limb is snapped off at approximately 45° to the skin. We found it beneficial to wind the limbs around the fingers such that only a small segment of suture filament is exposed above the skin to minimize the chance of mid-suture limb as opposed to at-the-knot breakage. Once the 3 sutures are broken, the sheath/dilator combination is immediately passed over the power lead and into the vasculature restoring hemostasis. Using this technique, knot breakage allowing advancement of the sheath/dilator into the vasculature was accomplished in all cases during the feasibility study.

#### Vessel closure

Once the Aortix pump has been removed, the large-bore hemostatic sheath remains in the vasculature. Then, the artery is closed using a modified dual-sheath procedure, modified by the fact that the 1-o’clock suture effectively becomes the first of the 2 vessel-aligned sutures placed during a dual-sheath or wire closure.

**Figure 7:**
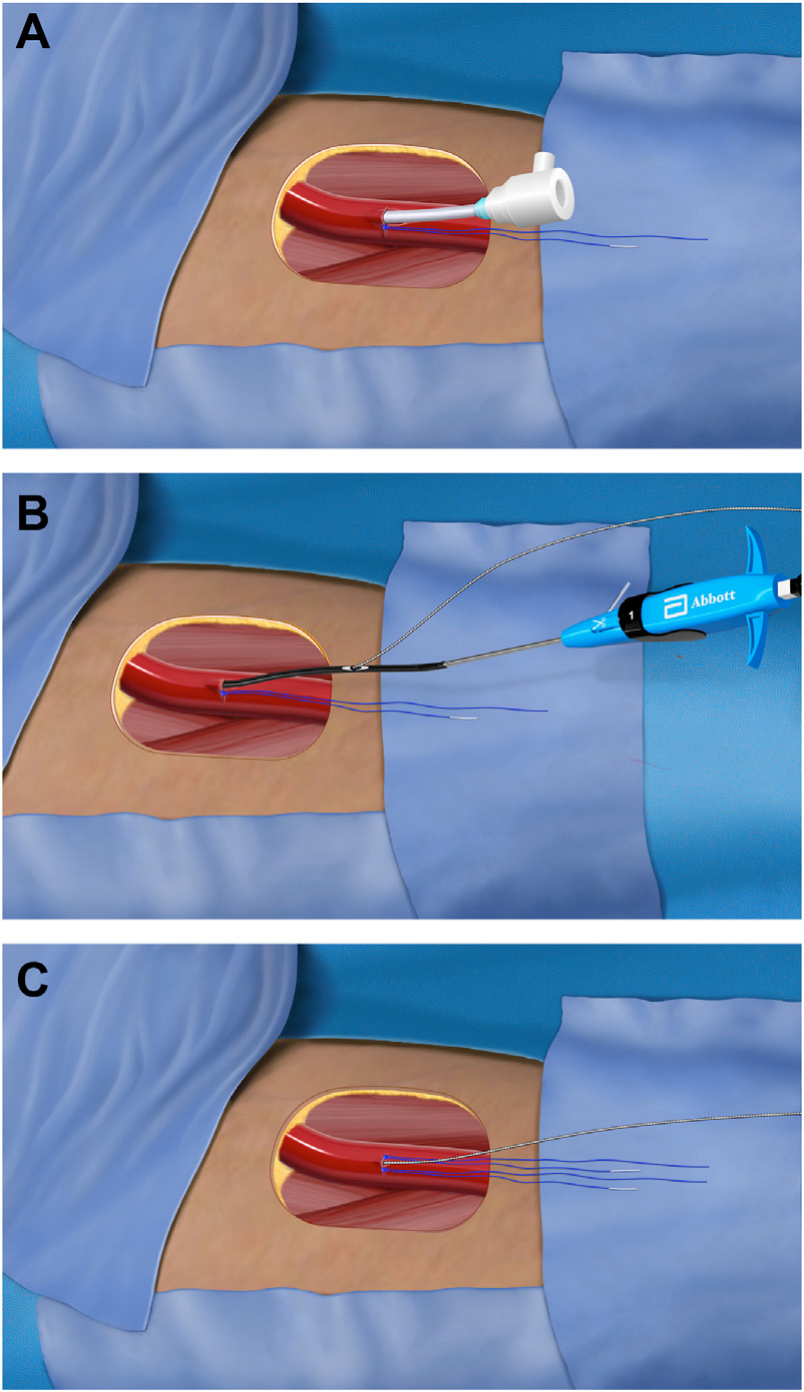
**Overview of the modified dual-sheath closure.** (**A**) The 1-o’clock in situ suture has been retraveled and locked. (**B**) A postclose Perclose is placed over a wire placed in the vessel through the 10F or 11F sheath. (**C**) The postclose Perclose is deployed and hemostasis assessed before removing the wire.

In the modified technique, an exchange length access wire is placed into the retrieval sheath. The retrieval sheath is then exchanged for a 10F or 11F standard access sheath placed over the wire while manual hemostasis is maintained. Tension is applied to the blue limb of the remaining in situ 1-o’clock suture originally placed in a preclose manner to begin terminal closure. Generally, this suture will be very effective at reducing bleeding because it was originally placed across an approximately 6F access in the artery. After retraveling the 1-o’clock suture, it may be left unlocked or immediately tightened and locked per the operator’s preference. Subsequently, the 10F or 11F sheath is exchanged for a new Perclose instrument to place an additional suture at the 12-o’clock position. This suture is tautened and traveled as usual, wire access is regained through the Perclose instrument, and the just-placed suture is traveled with blue limb tension and hemostasis assessed (Figure 7). The operator may remove the wire if hemostasis is adequate or elect to place a third Perclose also aligned to the 12-o’clock position. Alternatively, a collagen-based small-bore collagen closure device may be placed in lieu of or in addition to the third Perclose suture if desired.

Although the overall terminal closure paradigm is based on the original preclose 1-o’clock suture being available and effective to begin arteriotomy wall approximation, we are always prepared to perform a regular dual-sheath or wire closure if it is not.^2^ In such a case, we place 2 wires in the 10F to 11F sheath and alternatingly place 2 or more postclose sutures aligned with the vessel axis (12 o’clock) while the sheath is in the artery to force the suture to the outside of the arteriotomy. After suture deployment, we do not trim the suture limbs until hemostasis is assured because having intact limbs allows one to break and replace an ineffective suture.

As in other large-bore procedures, if necessary, dry closure can be performed using a noncompliant balloon passed across the bifurcation from the contralateral femoral artery or radial approach and used for tamponade while hemostasis is obtained. A further alternative is to use a nonsuture–mediated device specifically designed for large-bore closure. We used the Manta vascular closure device (Teleflex) successfully in 8 cases to close the 21F access site without concomitant use of Perclose.

Development of the methods described originated from learnings during the first 3 Aortix patients implanted in the feasibility study, which were implemented thereafter. After Aortix device placement using the above-described preclose methods, intermediate hemostasis was successfully obtained over the exiting power lead in 17 of the 18 patients. In 1 patient, operator difficulty with deployment of multiple Perclose devices placed before implanting the pump was causal and the patient underwent surgical closure.

In 8 patients implanted in the study, terminal closure after retrieval of the Aortix device was performed with a Manta device, but as the study progressed, suture-based terminal closure became the method of choice. Using the techniques described in this study, suture-based terminal closure was performed successfully in 11 of the 12 cases. In the 1 unsuccessful case, the implanter elected to not preclose a fourth suture at the time of implant to be available during terminal closure and closed using a covered stent. Learnings from that case were used to develop the above-described technique for use when no unlocked preclosed sutures are available for terminal closure and used in the remainder of the cases.

## Discussion

Vascular complications associated with the use of MCS devices are a major source of morbidity and mortality.^8^ Advances in percutaneous techniques and vascular access management remain essential to preventing major complications.^8^^,^^9^ Variability of vascular access and closure techniques among operators is common and may contribute to the high rate of complications seen.^10^ To reduce complications and improve outcomes, the use of “vascular safety bundles” to standardize access and closure techniques has been proposed.^10^ In our experience, standardizing the technique for suture-mediated closure in the Aortix procedure has been a key component to procedural success.

We described using 4 preclosed Perclose sutures in the Aortix pMCS access and closure technique although we recognized that many large-bore access sites are closed with 2 preclosed sutures after sheath removal.

There are several fundamental teachings/observations that led us to settle on 4 sutures. First, once the deployment sheath is removed, the 6F power lead remains in the vasculature and additional postclosed Perclose sutures cannot readily be placed should hemostasis not be achieved. Second, hemostasis is achieved by purse stringing around the relatively slippery polyurethane power lead; in our experience, although 2 sutures may provide adequate intermediate hemostasis on the table, patient motion during therapy may affect hemostasis. Three sutures have proven robust against the challenge of patient motion.

The fourth suture preclosed and unlocked in our technique serves to begin arterial wall approximation after retrieval sheath removal in the modified dual-sheath terminal closure technique; it may also serve as a backup suture to obtain intermediate hemostasis should one of the other 3 sutures prove inadequate.

The use of a suture-based preclose technique is the safest and most reproducible large-bore closure technique. Suture-mediated closure has been demonstrated to be superior to a current plug-based closure for large-bore access, albeit this has yet to be studied in patients receiving pMCS.^11^ In the Aortix procedure, suture-mediated closure is used in a novel fashion to both achieve intermediate hemostasis over the exiting 6F power lead and again later for terminal closure. Although many of the steps described in this study are familiar to the operator proficient with suture-mediated closure, key steps in the procedure, such as tautening and traveling of the suture and suture breaking, are not always performed in current practice. Tautening and traveling after suture release removes the slack from each suture after placement and enhances procedural success and is recommended whenever these devices are deployed. Retraveling of the suture knot to the artery using blue limb railing after sheath removal may additionally serve to reduce periprocedural bleeding by keeping the artery well apposed during serial dilation of the artery and sheath exchanges. The routine performance of tautening and traveling immediately on suture release may be applicable to other suture-based closures and assist with hemostasis in cases with indwelling large-bore devices, such as pMCS devices and extracorporeal membrane oxygenation cannulas.

The suture breaking technique, developed for reentry of the artery over the exiting power lead, is another technique that may have broader utility in practice. When proficient with this technique, operators may be comfortable in breaking sutures when it seems a suture is not effective, particularly if limited white rail shortening is observed while attempting to travel the knot.

## Conclusion

Using the techniques described, suture-mediated closure is a reproducible means of obtaining intermediate hemostasis during therapy with the Aortix device and terminal closure after pump retrieval. This technique uses a combination of preclose and postclose sutures. Specific procedural steps and order of operations may also prove to have value in other large-bore percutaneous procedures. The steps key to success in using multiple sutures are shown in the Central Illustration

**Central Illustration:**
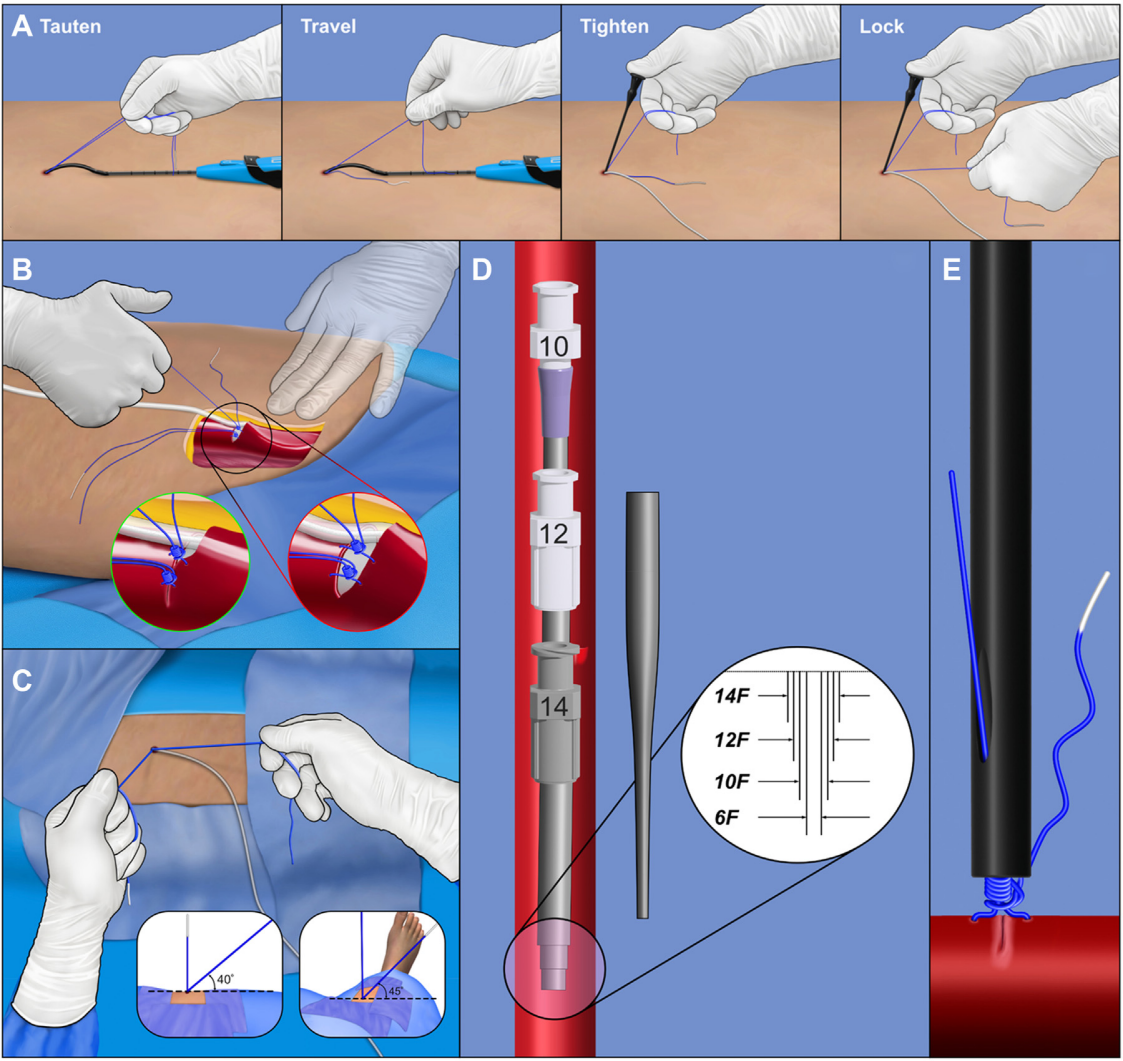
Key steps in deployment of multiple Perclose sutures in Aortix. (**A**) Certain key steps in obtaining successful vessel access and closure using the Aortix pMCS include the need to specifically perform the 4 Perclose deployment steps; (**B**) avoid excess pressure on the artery while retraveling the suture knot to allow optimal knot travel and arterial wall approximation; (**C**) ensure the hand position angles are as shown, before breaking a locked suture; (**D**) perform serial dilation in small sequential steps before placing the large-bore sheath; and (**E**) preferentially use the polymeric Snared Knot Pusher for increased proprioception and central pressure on the knot when tightening and locking the suture.
